# The Interaction of Diabetes and Tuberculosis: Translating Research to Policy and Practice

**DOI:** 10.3390/tropicalmed6010008

**Published:** 2021-01-08

**Authors:** Reinout van Crevel, Julia A. Critchley

**Affiliations:** 1 Department of Internal Medicine and Radboud Center for Infectious Diseases, Radboud University Medical Center, 6500HB Nijmegen, The Netherlands; 2 Centre for Tropical Medicine and Global Health, Nuffield Department of Medicine, University of Oxford, Oxford OX3 7LG, UK; 3 Population Health Research Institute, St George’s, University of London, London SW17 ORE, UK; jcritchl@sgul.ac.uk

**Keywords:** diabetes, tuberculosis, latent tuberculosis

## Abstract

Diabetes Mellitus increases the risk of developing Tuberculosis (TB) disease by about three times; it also doubles the risk of death during TB treatment and other poor TB treatment outcomes. Diabetes may increase the risk of latent infection with *Mycobacterium tuberculosis* (LTBI), but the magnitude of this effect is less clear. Whilst this syndemic has received considerable attention, most of the published research has focussed on screening for undiagnosed diabetes in TB patients or observational follow-up of TB treatment outcomes by diabetes status. There are thus substantial research and policy gaps, particularly with regard to prevention of TB disease in people with diabetes and management of patients with TB–diabetes, both during TB treatment and after successful completion of TB treatment, when they likely remain at high risk of TB recurrence, mortality from TB and cardiovascular disease. Potential strategies to prevent development of TB disease might include targeted vaccination programmes, screening for LTBI and preventive therapy among diabetes patients or, perhaps ideally, improved diabetes management and prevention. The cost-effectiveness of each of these, and in particular how each strategy might compare with targeted TB prevention among other population groups at higher risk of developing TB disease, is also unknown. Despite research gaps, clinicians urgently need practical management advice and more research evidence on the choice and dose of different anti-diabetes medication and effective medical therapies to reduce cardiovascular risks (statins, anti-hypertensives and aspirin). Substantial health system strengthening and integration may be needed to prevent these at risk patients being lost to care at the end of TB treatment.

## 1. Introduction

Almost a hundred years ago, some clinicians observed and reported an association between diabetes mellitus and tuberculosis. Insulin was introduced in 1922, and of those type 1 diabetes patients who did not die from diabetic coma, many were thought to die from tuberculosis. In 1934, Howard Root, a physician from Boston, used autopsy studies to conclude that juvenile diabetes was associated with a 10-fold increased risk of tuberculosis, mostly occurring within years following recovery from diabetic coma. Tuberculosis was also more common in adults with diabetes, following the onset of diabetes in 85% of cases [1]. Joint TB and diabetes treatment clinics were even established in Birmingham, UK around this time period to try to improve the very poor outcomes for people with TB–diabetes [2]. With declining tuberculosis incidence and mortality, and the introduction of anti-tuberculous drugs from 1944/1945 onwards, this relationship was somewhat forgotten. Then, in the 1990s as prevalence of type 2 diabetes started to rise substantially in many low- and middle-income countries where TB remained endemic, a number of epidemiological studies “rediscovered” diabetes as a risk factor for tuberculosis [3,4,5]. However, this was overshadowed by the emergence of the HIV epidemic which reversed downward trends in tuberculosis incidence and deaths. However, following more systematic reviews on the link between these two diseases, [6,7] the Global Tuberculosis Union and the WHO developed a collaborative framework for tuberculosis and diabetes in 2011, and in 2015 the TB Union and the World Diabetes Foundation hosted a summit in Bali and issued the “Bali declaration” that advocated implementation of this framework and further research to address the looming convergence of diabetes and tuberculosis globally [8].

Many studies have been conducted on the link between diabetes on tuberculosis. In this review, we focus on research findings that have practical relevance for policy and clinical practice. This also includes our experiences and published and sometimes unpublished findings from the EU-funded TANDEM (tuberculosis and diabetes mellitus) program that combined field studies in four tuberculosis-endemic countries (Indonesia, Peru, Romania and South Africa) and laboratory sciences to study clinical and pathophysiological aspects of combined diabetes and tuberculosis [9]. We address four topics: epidemiological effects of diabetes on tuberculosis; screening strategies for combined disease; combined treatment of diabetes and tuberculosis; and possible control of diabetes-associated tuberculosis at a population level. Wherever possible, we refer to the guidebook on combined diabetes and tuberculosis issued by the TB Union and the World Diabetes Foundation [10].

## 2. Epidemiological Effects of Diabetes on Tuberculosis

The global epidemic of diabetes mellitus, affecting approximately 425 million individuals in 2017 and estimated to grow to 629 million people in 2045, has enormous medical and social consequences [11]. More than 80% of type 2 diabetes mellitus, referred to as “diabetes” in this review, is found in low- and middle-income countries and in areas where TB remains endemic. Diabetes is often undiagnosed and is also frequently complicated by cardiovascular complications and eye, foot and kidney problems. 

Diabetes also increases the risk of many infections and their complications [12], and a serious infection has often been the first presentation of diabetes in many settings. Infections in people with diabetes are common and some infections are so strongly associated with diabetes (“signal infections”) that they are rarely seen in people without diabetes, at least in high income countries [13]. The current COVID-19 pandemic has reinforced the increased risk of severe infections in people with diabetes [14], but recent evidence suggests type 2 diabetes increases risks of needing hospital treatment for any infection by about two times, and type 1 diabetes by nearly four times [15]. Almost all of the studies of TB and diabetes from low- and middle-income countries (LMIC) have been of type 2 diabetes, and hence there is a lack of data on the possible effects of type 1 diabetes on TB in these settings. 

### 2.1. Diabetes and Natural History of TB 

**Figure 1:**
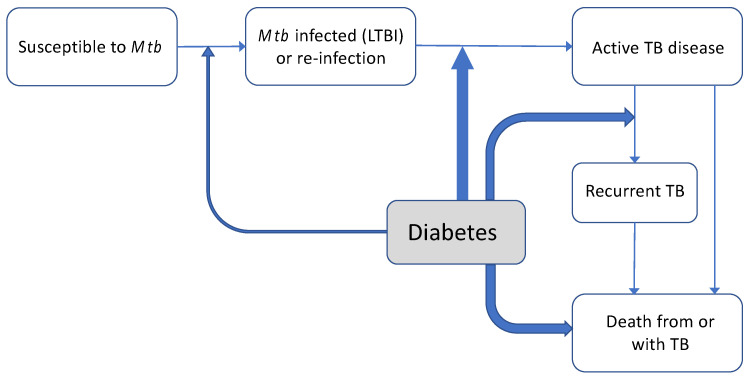
Effects of diabetes on natural history of tuberculosis. Natural history of TB, with the impact of concomitant diabetes at various points on this pathway depicted. The width of lines represents current understanding of magnitude and certainty of evidence (see references [18−20,24,27] in text for evidence for and magnitude of possible relationships between diabetes and TB susceptibility, TB disease and prognosis).

The natural history of TB is complex and there are several different points along the pathway to disease where diabetes might be increasing TB risk (see Figure 1). Many people clear *M. tuberculosis* infection on initial exposure, but others will become latently infected and remain at risk of future development of TB disease. It is estimated that around 25% of the world’s population have latent TB (LTBI), and many of them already have or are at risk of developing diabetes as well. An estimated 5% of infected individuals will progress to active TB disease over their lifetime, about half of those within 1–2 years after becoming infected (rapid progression).

Observational studies (cohort studies) usually cannot clearly distinguish whether a higher risk of TB disease among people with diabetes might be driven by an increased risk of infection with *M. tuberculosis* in people with diabetes (LTBI), from an increased risk of developing TB disease in people who have LTBI infection or both. It is generally thought that increases in TB disease among those with LTBI might be more important than a markedly increased propensity for infection with *M. tuberculosis* in the first place (see Figure 1). A recent cross-sectional study from Bandung, Indonesia (n = 682 diabetes patients) supported this view; whilst the prevalence of LTBI was lower in Indonesian diabetes patients than in a comparator group of household contacts (LTBI: 38.6% vs. 68.6%), people with diabetes were much more likely to have TB disease: 4.9% vs. 1.2% in diabetes patients compared with household contacts. One study from a low TB incidence country (Denmark) found an increased risk of TB disease only in people within a few years (two years) of diabetes diagnosis and not for those with diabetes of longer duration [16]. Some have argued this might suggest a role of diabetes (and particularly initial hyperglycaemia) in progression from LTBI to active disease, but this could also reflect diagnostic bias. At present, there have not been sufficient large-scale longitudinal studies or whole genome sequencing studies that could help elucidate the mechanisms involved more clearly [17].

### 2.2. Diabetes and Risk of M. tuberculosis Infection

However, increased exposure to *M. tuberculosis* and hence higher infection risk might also be present in people with diabetes. This could potentially occur through increased use of health care facilities or as a result of immune system alterations in people with diabetes, making initial infection more likely. A recent systematic review found only a modest increased risk of *M. tuberculosis* infection in people with diabetes (RR 1.18, 95% CI 1.06 to 1.30) [18]. However, most of the studies included in this review were cross sectional in design and had significant limitations. In particular, few of the studies included used laboratory methods to screen for undiagnosed diabetes and most included only those with known diabetes in the exposure category, resulting in misclassification of diabetes and potentially underestimating the risk of LTBI associated with diabetes [19]. One recently published US cross-sectional study screened all refugees for diabetes using glycated haemoglobin (HbA1c). This showed higher infections risks and a graded response, with increased risk of LTBI among people with “pre-diabetes” (HbA1c 5.7–6.4%; OR 1.7, 95% CI 1.1–2.4) and higher among those with diabetes (OR 2.3, 95% CI 1.2–4.5) [20] A community based study from Taiwan with more robust case and outcome definitions also identified an increased risk (OR: 1.59; 95% CI 1.11 to 2.28) [19]. Cohort studies following patients with newly diagnosed diabetes to determine LTBI status would improve understanding of the magnitude of *M. tuberculosis* infection risk and potential mechanisms but are obviously difficult to carry out in LMIC.

### 2.3. Intermediate Hyperglycaemia and TB

Pre-diabetes (sometimes termed non-diabetes dysglycaemia or intermediate hyperglycaemia (IH)) is rising rapidly in many parts of the world, particularly in areas where TB is endemic. The IDF Diabetes Atlas estimated that about 7.5% of adults aged 20–79 had impaired glucose tolerance (IGT; based on an oral glucose tolerance test) in 2019, but this figure might be substantially higher if patients with impaired fasting glucose or elevated HbA1c were also included in the definition, [21,22] potentially up to 1/3 of adults in many countries. There are relatively few robust studies investigating links among IH, TB infection, disease and poor treatment outcomes but limited evidence suggests these could also be somewhat elevated [20]. Even relatively small increases in risk could be important at a population level given the high prevalence of the condition. A recent study also identified higher bacterial loads among newly diagnosed TB patients with IH in India, highlighting the potentially greater risk of transmission also [23]. 

### 2.4. Effect of Diabetes on TB Disease Risk, Presentation and Treatment Outcomes 

Whatever mechanisms are involved, several systematic reviews have now summarised evidence from observational studies (particularly cohort studies, where the temporality of the exposure and outcome is more clearly established) showing that the risks of TB disease are significantly elevated in people with diabetes, about 2–3 times higher. [6,7,24].

Many studies have also found that diabetes alters the presentation of TB, with more cavitation, higher severity TB scores and more smear or culture positive pulmonary TB [25]. Extra-pulmonary TB disease appears to be less common in people with diabetes, although this is contested [6,7]. However, this pattern is very different from patients with HIV co-infection, which very clearly increases presentation with extrapulmonary and disseminated TB [26]. Diabetes may also slightly increase the bacterial load of *M tuberculosis* and lengthen the time to smear or culture negativity; nearly twice as many patients with TB and diabetes remain culture positive at Months 2–3 compared to those with only TB. Many studies and reviews have now demonstrated that diabetes worsens TB treatment outcomes, in particular doubling the risk of death during TB treatment [27]. Cause of death during treatment among people with TB and diabetes is not always clearly reported, but not only diabetes but also TB is associated with an increased risk of cardiovascular complications such as myocardial infarction [28,29] and stroke [30], possibly explaining the higher rate of deaths in the first few months of TB treatment in patients who also have diabetes [31,32]. Even after TB treatment success TB patients remain at increased mortality risk, possibly due to increased cardiovascular risk regardless of whether or not they have diabetes [28,33,34], although most studies have significant limitations (including limited follow-up time, lack of control of key confounders, or inappropriate selection of control series) [35].

Diabetes seems to increase the risk of TB recurrence and also appears to be associated with a doubling of the risk of identification of multi-drug resistant TB [36]. Poorly controlled diabetes (as measured by high HbA1c or fasting blood glucose) is associated with increased TB susceptibility and might also worsen TB treatment outcomes (see Figure 1). Although evidence is still limited, as shown in a recent systematic review [37], better diabetes control could reduce some of these risks [38,39,40]. In patients with dual disease, there is also evidence that poor lifestyle practices (e.g., the continuation of cigarette smoking in people with diabetes) can synergistically increase risk of TB disease and poor outcomes [32,41,42] Thus, clearly, clinical management of combined TB and diabetes is important, despite the lack of attention it has received in the research agenda to date.

## 3. Screening and Diagnosis of Combined Diabetes and Tuberculosis

Screening of newly registered TB patients for diabetes is recommended by WHO guidance and other national bodies and has been incorporated into DOTS management for TB in many countries, e.g., India, although this may not always be performed well in practice [43]. International guidance suggests that screening could be achieved through a simple blood glucose or glycated haemoglobin test [10], although it is thought that about half of TB clinics globally do not have access to laboratory facilities, even for blood glucose measurements [44]. Point of care tests may thus be useful, particularly in recognising very elevated levels of blood glucose where specialist input into care might be warranted, but they are less accurate and thus need careful interpretation [36]. Any screening should ideally be repeated later in TB treatment, when transient hyperglycaemia is likely to have resolved. In the absence of any screening facilities, simple risk scores including variables such as anthropometry, family history of diabetes, diagnoses and treatment of hypertension and physical activity levels have been developed among TB patients and could be useful in identifying undiagnosed diabetes, but they are of lower accuracy and require further validation studies [44]. There may be significant heterogeneity in the accuracy of any screening strategy, dependent on the local epidemiology of diabetes, extent of hyperglycaemia and the prevalence of undiagnosed diabetes, as well as the efficiency of the TB system. All of these will significantly affect the positive predictive values [44], yield and cost-effectiveness of screening TB patients for diabetes in different settings.

Screening of diabetes patients for active TB has been advocated by some since theoretically it might contribute to case detection and thus TB elimination, but the yield of TB cases is likely to be quite low in most settings, except where TB incidence is already known to be very high. A recent study in South Africa screened 440 diabetes patients for TB using sputum culture and GeneXpert, finding that 3% (95% CI 1.72–5.03) of a cohort of diabetes patients had TB, half of these with no symptoms [45]. Active case detection in Myanmar [46] and in Bandung, Indonesia [47] also identified TB in about 2% and 1.5% of patients, respectively. Studies elsewhere have mostly found few or no cases of active TB [48,49]. Most of these studies relied on active case detection in people with symptoms, and thus might potentially miss some cases, but it seems unlikely that such systematic screening is cost-effective [50]. Focussing any screening on diabetes patients at highest risk (identified by low BMI, poorer diabetes control, low triglyceride levels or older age, as well as smoking status [47]) may be more feasible but requires further investigation. Recent guidance does not therefore promote systematic or routine screening of diabetes patients for TB [10]. Rather, a focus on screening following initial diabetes diagnosis and increased physician and patient awareness of risks and symptoms seems warranted.

Some key issues related to screening for combined diabetes and tuberculosis are shown in Table 1, and recommendations have been included in the recent guideline on TB and diabetes [10].

## 4. Treatment of Combined Diabetes and Tuberculosis

As previously highlighted, diabetes both increases susceptibility to tuberculosis and leads to worse treatment outcomes, with more deaths, tuberculosis treatment failures and recurrent disease. This raises the question if and how clinical management of combined diabetes and tuberculosis can be optimised. There is relatively little evidence to guide clinicians in clinical management of combined disease. We separately address tuberculosis and treatment and diabetes management in people with combined disease. Some key issues related to treatment of combined TB and diabetes are included in Table 2, and recommendations have been included in the recent guideline on TB and diabetes [10].

### 4.1. Tuberculosis Treatment in Patients with Comorbid Diabetes

Currently recommended TB treatment is similar for patients with combined TB and diabetes compared to those with TB only. However, this may have to be reconsidered as diabetes is associated with TB drug resistance, [51] slower treatment response and higher rates of toxicity, failure and recurrent TB. First, TB treatment might have to be adjusted in length, as seems common practice already in some countries, including China [52]. Indeed, in a large retrospective cohort study from Taiwan, nine-month treatment was associated with a lower rate of recurrent TB than six-month treatment (hazard ratio 0.76; 95% CI 0.59–0.97) [53].

Besides length of treatment, higher dose TB treatment may also help improve treatment outcomes. Some studies have reported associations between diabetes and lower concentrations of TB drugs, [54,55] although this may partly be explained by inappropriate correction for body weight in TB patients with diabetes who are generally significantly heavier than those without [56]. However, in an observational study in the USA, therapeutic drug monitoring for INH and rifampicin after two weeks treatment was associated with significantly shorter time to sputum culture conversion among patients with combined TB and diabetes (42 versus 62 days; *p* = 0.01) [57].

Another factor to consider is the apparent association between diabetes and TB drug resistance. A recent meta-analysis included 9289 patients from 13 studies found a significant association between diabetes and multi-drug resistant (MDR)-TB (OR 1.71, 95% CI 1.32–2.22) [27,51]. Possible factors contributing to this association include higher rates of acquisition of drug-resistance with higher initial bacterial load or slower response to treatment or nosocomial acquisition of drug-resistance with higher rates of hospital admission. However, diabetes is also associated with *primary* drug resistance. TANDEM performed the first study to compare genotypic drug resistance between TB patients with (n = 159) and without diabetes (n = 737) [58]. This study used whole genome sequencing on an unselected cohort of patients from Peru and all TB–diabetes plus age-matched non-diabetic TB patients from an Indonesian cohort. Drug resistance mutations were found in isolates of 18% of diabetic and 15% of non-diabetic patients in Indonesia and 39% of diabetic and 20% of non-diabetic patients in Peru, with *rpoB* (rifampicin)*, fabG1* (INH) and *gyrA* (fluoroquinolone) mutations associated with diabetes. In multilevel multivariable logistic regression, diabetes was the only factor significantly associated with genotypic drug resistance against at least one drug (OR 1.8, 95% CI 1.1–2.9). Interestingly, the association between diabetes and drug resistance was similar for patients with new (adjusted OR 2.0, 95% CI 0.9–4.4) and previously diagnosed diabetes (OR 1.7, 95% CI 0.98–2.9). This suggests that individuals with diabetes may be more susceptible to drug resistant *M. tuberculosis* isolates; some drug resistance mutations lead to “loss of fitness” of *M. tuberculosis,* but such “less fit” strains might still cause active tuberculosis among individuals with diabetes because of their decreased host immune function against tuberculosis [59]. Whatever the underlying cause, TB–diabetes patients should probably be prioritised for drug susceptibility testing (DST) in those settings where DST is not routinely done for all patients, both at baseline and during follow-up. The Xpert MTB/RIF assay (Cepheid Inc, Sunnyvale, CA, USA) is being scaled up globally and within 2 h allows the confirmation of *Mycobacterium tuberculosis* and the detection of rifampicin resistance—which is equivalent to MDR-TB. In 2013, WHO recommended that the assay be considered as the initial diagnostic test for all people requiring investigation for TB. While this is yet to happen for all patients, those with TB–diabetes should be prioritised.

There are several other things to consider when treating patients with combined diabetes and TB. For instance, there is a higher risk of drug–drug interactions. Rifampicin increases the metabolism of many drugs commonly used by diabetes patients including statins, all oral diabetes drugs except metformin, calcium-channel blockers, ACE-inhibitors, digoxin and warfarin. some antihypertensive drugs (van Crevel, Tuberculosis, in Cohen and Powderly, 2017). In addition, TB–diabetes patients may have more symptoms or symptoms that are more difficult to interpret. For instance, abdominal symptoms in an elderly TB patient with diabetes may be a side effect of TB drugs, hepatotoxicity, side effects of metformin, inferior myocardial infarction or bowel ischemia. In addition, drug toxicity may be worse; INH can aggravate diabetic neuropathy, and TB–diabetes patients have a higher risk of liver and kidney toxicity. This is also relevant for management of MDR-TB; patients with diabetes probably have a higher risk of renal toxicity with aminoglycosides and a higher risk of neuropathy with linezolid. Furthermore, a high pill-load when patients are treated for both diseases may lead to missed doses, incorrect drug intake, treatment interruptions or default. These and other issues necessitate more careful assessment prior to and during combined TB and diabetes treatment. It should be stated that there is a scarcity of data regarding all these aspects and that more study is needed.

A final consideration may be HIV co-infection; Sub-Saharan countries with a high TB–HIV burden are witnessing the most rapid growth in diabetes prevalence, while some other countries where TB–diabetes is common (e.g., India or Indonesia) show significant growth of the HIV epidemic. HIV is associated with diabetes; in a study in Tanzania, glucose metabolism disorders were six-fold more common among HIV-infected individuals compared to age- and weight-based controls [60]. In addition, in Ethiopia and South Africa, longer duration of HIV treatment was associated with increased incidence of diabetes [61,62]. Finally, in part due to the successful roll-out of antiretroviral treatment, increasing numbers of HIV-infected patients are surviving to older ages and become at increased risk of developing diabetes [63]. Therefore, a growing number of people may be affected by diabetes, HIV and active TB at the same time. In such cases, there is an even higher risk of drug–drug interactions, toxicity and overlapping side-effects, for which clinicians will need guidance.

### 4.2. Optimising Diabetes Management in Patients with Combined Tuberculosis

Management of diabetes is aimed at reducing short- and long-term complications such as cardiovascular disease, eye and kidney problems and foot amputations. Diabetes management consists of: lifestyle counselling (diet, weight loss, physical activity, smoking cessation and avoiding excess alcohol); treatment with blood glucose lowering drugs; measures to reduce the risk of cardiovascular disease and associated complications that include anti-hypertensive medications, lipid-lowering drugs and anti-platelet drugs if indicated; and management of specific complications such as diabetic feet and eye problems. The first priority for patients with combined diabetes and TB is to successfully initiate TB treatment, but diabetes management certainly deserves attention. For instance, severe hyperglycaemia, which can be symptomatic and is likely to affect TB outcomes, should be treated. TB itself may also increase risks of cardiovascular disease, as previously noted [35].

As described above, TB–diabetes patients form a heterogeneous group, consisting of those with “known” (previously diagnosed) and newly diagnosed diabetes, with hyperglycaemia ranging from mild to severe, variable duration of disease and highly variable comorbidity, disease complications and treatment needs. Around 74% of TB–diabetes patients in the TANDEM cohort in Indonesia, Peru, Romania and South Africa had previously diagnosed diabetes, while 26% were newly detected as a result of diabetes screening. Few patients with “known” diabetes were still under diabetes care when their TB was diagnosed, some had never even been started on diabetes treatment [64] and those supposedly taking medication showed very poor glycaemic control and often high cardiovascular risk. TB–diabetes patients also vary in terms of the number of diabetes complications and cardiovascular or other co-morbidities. Heterogeneity of diabetes in TB patients is especially notable between different countries, most likely due to genetic, environmental, nutritional and behavioural factors and differences in accessibility and quality of health services. Clearly, this heterogeneity has important implications for diabetes management.

### 4.3. Glycaemic Control

An accepted target for glucose control in diabetes is an HbA1c < 7% (53 mmol/mol), as recommended by the American Diabetes Association [65], although the American College of Physicians recently recommended HbA1c levels of 7–8% [66]. However, this may be hard to achieve during anti-TB treatment, due to drug interactions with rifampicin and altered patterns of food intake and energy expenditure during TB disease and treatment recovery [25]. Advanced or long-standing diabetes may also render better control more complex. Some randomised controlled trials of “tight control” (target < 6% HbA1c) among older people with long-standing type 2 diabetes in high-income countries had unexpected, unfavourable outcomes [67]. Therefore, although this is not entirely evidence-based, a more realistic and cautious treatment target—especially under programmatic conditions—may therefore be HbA1c < 8% and a target of RBG/FBG < 11.1 mmol/L (200 mg/dL) during the treatment of TB disease, which is in line with those for diabetes management in persons with significant co-morbidity [65]. It is also important to realise that TB-associated inflammation can lead to temporary hyperglycaemia, which often spontaneously improves with anti-tuberculosis treatment [68]. For instance, a Tanzanian study found that associations between hyperglycaemia and TB present at baseline were substantially attenuated after TB treatment; however baseline hyperglycaemia was associated with substantially increased risk of death or TB treatment failure) [68]. Not all studies have shown such significant reversions of hyperglycaemia; this occurred in only 3.7% of patients in Indonesia [69] and only 14% of TB patients with newly identified hyperglycaemia in the TANDEM study, which unlike many other studies used repeated tests to confirm hyperglycaemia increasing the certainty of the initial diagnosis. Hyperglycaemia in patients with “known diabetes” proved much more difficult to reduce in a small trial of improved diabetes management during TB treatment than among those with “new diabetes” (Koesoemadinata et al., under review).

The next question is how optimal glycaemic control can be achieved. Thus far, there is no guiding evidence as to what drugs should be used, how treatment should be monitored, who should best deliver diabetes treatment and how this should be adjusted for patients with new or previously known diabetes and for those with mild or severe disease. We first address the choice of glucose-lowering drugs, discussing three classes of drugs: biguanides, sulphonyl urea derivates and insulin. Although there are other drug classes to treat diabetes, including thiazolidinediones (TZD), DPP-4 inhibitors, SGLT2 inhibitors and GLP-1 receptor agonists, these medicines are more expensive and there is limited evidence of superior effectiveness.

Metformin is the first choice glucose-lowering agent recommended in type 2 diabetes, and there is no reason this should be different for patients with active TB disease. The advantages of metformin include extensive experience with the drug, extremely low risk of hypoglycaemia, effectiveness, low cost, beneficial effects on cardiovascular disease, [67] lack of clinically relevant interaction with rifampicin [70] and a potential benefit on TB itself [71]. In a recent retrospective analysis from Taiwan, those with diabetes (30%) had a 1.9 times higher mortality, but among this group, metformin use was associated with lower mortality (HR 0.56, 95% CI 0.39–0.82) [72]. However, all data on metformin’s effect on TB is from observational datasets and might be affected by selection biases, as metformin is the first line treatment for diabetes in many parts of the world. Diabetes patients taking other drug treatments may thus be substantially different in ways that affect TB prognosis and are difficult to adjust for. Metformin’s two main disadvantages are gastro-intestinal side effects, which may be worse when taken together with TB drugs (TANDEM, unpublished) and increased toxicity, including the development of lactic acidosis in patients with decreased kidney function. Lactic acidosis, usually presenting as vomiting, abdominal pain, signs of hypovolemia followed by Kussmaul breathing, neurological signs, cardio-respiratory, kidney or liver failure and finally death is extremely rare but it may be fatal if unrecognised and untreated [73]. The dose of metformin needs adjustment with a renal clearance (eGFR) < 50 mL/min.

Sulphonyl urea derivates can be used as second choice oral glucose-lowering agents, probably as “add-on” to metformin if metformin alone is ineffective or if there is intolerance or a contraindication to metformin. The most widely used sulphonyl urea derivates are gliclazide, glibenclamide, glimepiride and glipizide. The two main disadvantages are the risk of hypoglycaemia and strong drug interactions with rifampicin that show wide inter-individual variation but result in their efficacy being reduced by 30–80% [74].

Some argue that insulin is the preferred glucose-lowering treatment for TB–diabetes patients, but especially under programmatic conditions insulin is probably the third choice, except for sick and hospitalised patients, or patients who were already using insulin prior to a TB diagnosis. Insulin is indicated in cases of severe hyperglycaemia (e.g., HbA1c > 10% or blood glucose > 18 mmol/L). It has unlimited efficacy, but it is more expensive, requires refrigeration and subcutaneous injection and is associated with a risk of hypoglycaemia. In well-resourced settings, the use of insulin is usually accompanied by the need for self-monitoring of blood glucose through glucometers. Unavailability, insecure supply or high costs of diabetes medication, as well as issues related to storage and use of insulin, all compromise glycaemic control in many resource-constrained settings [75,76]. 

### 4.4. Adjustment of Treatment According to Patient Characteristics and Local Circumstances

Disease phenotype and severity (both with regard to TB and diabetes) and local circumstances will guide choice, timing and dosing of glucose-lowering drugs. Patients who are under diabetes treatment at the time of TB diagnosis can mostly continue their medication, although worsening hyperglycaemia as a result of TB may require intensification (e.g., increased insulin dose). Oral glucose-lowering drugs may have to be substituted or adjusted as, except for metformin, their metabolism is increased and therefore their glucose-lowering effect is decreased with concurrent use of rifampicin.

In TB patients with newly or known yet untreated diabetes, choice and timing of glucose-lowering drugs depends on the level of hyperglycaemia and local circumstances. If hyperglycaemia is mild (e.g., a HbA1c < 8%), initiation of glucose-lowering drugs can probably be postponed for at least 2–8 weeks. Mild hyperglycaemia often disappears with TB treatment only, and early start of glucose-lowering treatment may jeopardise successful TB treatment because of side-effects, drug toxicity, pill burden or difficulty for practitioners and patients to focus on two diseases at the same time.

Most TB patients are managed in ambulatory care and monitored at weekly or longer intervals in community or hospital outpatient clinics. In the early phase of TB treatment, TB patients should preferably not be referred to specialised diabetes services because of the risk of transmission of *Mycobacterium tuberculosis* to those working in or attending these clinics. In addition, separate management of TB and diabetes has the risk of unrecognised drug interactions, medication errors and decreased retention to either TB or diabetes treatment. Thus, preferably, diabetes treatment is delivered at the TB clinic, as has been reported previously [77].

One question is how diabetes can best be managed in TB clinics. To achieve optimal glycaemic control during TB treatment, monitoring of blood glucose during the course of TB treatment may have to be more frequent. However, frequent monitoring is associated with additional costs, and tools and skills for glucose monitoring and diabetes treatment may be lacking in TB or pulmonary clinics. As such, a less intense schedule, preferably following the established decision points in TB treatment after two and six months, would offer significant advantage. To address this dilemma, the TANDEM project has conducted a randomised study in Indonesia to evaluate the effect of regular scheduled glucose monitoring and algorithm driven adjustment of diabetes medication on glycaemic control of diabetes in TB patients (NCT02106039). Among 150 TB–diabetes patients with a median baseline HbA1c > 11%, compared to standard care, use of a structured clinical algorithm led to much steeper decline of HbA1c (with a difference of 1.82% HbA1c; *p* < 0.001) and doubling the proportion of patients with an HbA1c < 8% at six months (Koesoemadinata, under review). This suggests good glycaemic control in TB–diabetes can be attained through a package of education and use of simple treatment algorithms.

### 4.5. Cardiovascular Risk Assessment and Management

Atherosclerotic cardiovascular disease—including myocardial infarction, stroke and peripheral arterial disease—is the leading cause of morbidity and mortality for individuals with diabetes. Cardiovascular risk assessment is focussed on four possible interventions: lifestyle counselling; antihypertensive treatment; lipid-lowering treatment (statins); and treatment to reduce platelet aggregation (aspirin). To our knowledge, there is no published evidence or experience with respect to cardiovascular risk assessment and management in TB patients with newly diagnosed diabetes, but based on expert opinion, the recent guidebook on TB–diabetes put forward a number of targets and related interventions. [10,78] Successful initiation of TB treatment is of much greater importance in patients with newly diagnosed TB and diabetes than assessment and management of cardiovascular risk. Therefore, the guideline states that only considerations in such cases would be initiation of aspirin for those with established cardiovascular disease (e.g., a history of stroke or myocardial infarction), unless patients suffer from considerable haemoptysis, and counselling for smoking cessation and reduction of alcohol consumption. After completion of the initial intensive phase of anti-TB treatment (usually at eight weeks), patients could be counselled about other healthy lifestyles, and antihypertensive medication and statin treatment could be started as indicated. This is explained in more detail in the recent guideline [10].

## 5. Control of Diabetes-Associated Tuberculosis at a Population Level

Much of the initial focus of action on the TB–diabetes syndemic has concentrated on screening, particularly for undiagnosed diabetes among TB patients, where the yield is often very high. This is logical since globally about 15% of newly registered TB patients have diabetes [79] and many are undiagnosed. Enhanced diabetes screening and management during TB treatment might improve TB treatment outcomes, as well as subsequently long-term health for people living with diabetes. There are suggestions from several observational studies that better diabetes management might reduce TB risk and improve TB treatment outcomes [40,80], although these studies may be affected by selection biases, and randomised controlled trial data are lacking. Screening for diabetes might therefore be clinically important for the individual newly diagnosed with TB. However, in routine practice, it is less clear that this approach results in improved longer term health outcomes for a variety of reasons. Some of these relate to the difficulties in maintaining continuity of care for patients who have completed TB treatment; DOTS care for TB is generally well organised and freely available, but in many settings health systems are vertically aligned, and patients with newly diagnosed diabetes completing TB treatment are not routinely referred to diabetes care. In general, diabetes management in the public sector can be poorly organised or too expensive for people with TB disease, who are often very disadvantaged socio-economically [81,82]. Anecdotal evidence suggests many TB patients newly diagnosed with diabetes are therefore lost to the health system after their TB treatment is completed (TB Alert India, personal communication).

More fundamentally, trying to improve management of diabetes after a patient has already developed TB is intervening rather “late” in the natural history of TB disease [17]. Earlier intervention, either preventing or reverting diabetes, or preventing development of TB disease among people living with diabetes, could be a much more powerful approach. In populations such as India where diabetes prevalence is very high and TB remains endemic, interventions targeted at preventing TB in people with diabetes have the potential to make a population impact, reducing TB incidence by possibly 10–20% overall [83]. Roughly, there may be four strategies (Table 3). Firstly, a TB vaccine that is more successful in preventing TB in adults would be the ideal intervention, with implications far beyond diabetes-associated TB. Second, better prevention and management of diabetes could also have a substantial population effect on TB incidence and mortality, and also would have many other benefits in reducing risks of vascular disease and other diabetes complications. However, diabetes prevention and improving diabetes management in low-income settings have both proved very challenging. The latter has substantial resource implications and requirements for training many health care providers, given the large numbers of patients with poorly managed diabetes [82,84]. A third TB prevention strategy could be latent tuberculosis infection (LTBI) screening and preventive therapy in people with diabetes; modern treatment regimens (e.g., 3HP) are shorter and simpler than previously used 6–9 months of isoniazid, making this approach more feasible. However, LTBI treatment may have costs and adverse effects on individuals with diabetes, many of whom would never go on to develop active TB disease. This is because diabetes patients are likely to be older, with more multi-morbidities and co-medication, increasing the risk of toxicity and side effects compared to other groups being treated with LTBI preventive therapy. There is also a potential population level risk from increasing drug resistance if active TB disease is not effectively screened and treated among such patients before commencing LTBI treatment. To fully assess the costs, risks and benefits, a large first randomised controlled trial of LTBI preventive therapy among diabetes patients (who are HIV negative) is being established in Uganda and Tanzania (PROTID; NCT04600167). On completion, it will be possible to compare the potential cost-effectiveness of targeting patients with diabetes for LTBI treatment with other population groups at higher risk of TB disease, such as migrants. There might also be significant difficulties scaling up LTBI preventive therapy to all diabetes patients with LTBI even if this were demonstrated to be safe and cost-effective. However, a more targeted approach, identifying patients at greatest risk of active TB disease and prioritising such groups for treatment (e.g., through low BMI, severe hyperglycaemia or long duration of diabetes disease) through both this trial and other on-going research might be feasible. Clearly, the Holy Grail of TB prevention would be to reduce the incidence of diabetes and even small reduction would have large benefits; TB programmes should thus also provide support and advocacy to encourage nations to implement sustainable and comprehensive global action plans for diabetes prevention.

## 6. Summary

Diabetes may be affecting the natural history of TB in many different ways, resulting in potentially increased risks of TB infection, clearly much higher risks of TB disease, and also poorer TB treatment outcomes, especially mortality both during and after the end of treatment. Whilst there are many points on this pathway where health systems can improve outcomes, earlier interventions to prevent the development of TB in this at risk patient group may be most effective from a population perspective. Screening TB patients for undiagnosed diabetes has a very high yield and should be done in all adult patients, using anamneses (as many patients have previously been diagnosed with diabetes) and HbA1c or glucose blood tests. Screening people with diabetes for TB may be considered in very high-endemic settings, potentially targeted at patients mostly likely to have TB disease (due to low BMI, older age, smoking or other risk factors) to improve cost-effectiveness. Chest X-ray examination may also be a helpful triage instrument to select patients for microbiological testing. With regard to treatment, there are many challenges and substantial evidence gaps with regard to combined management of TB and diabetes, as well as combined TB, diabetes and HIV. TB treatment may have to be intensified, and special attention is required for higher rates of drug resistance. Optimal targets and treatment algorithms for diabetes management in TB patients still need to be defined, both with regards to glycaemic control and cardiovascular disease (risk). We also have to define what is needed in terms of optimal health service delivery, preferably in tuberculosis clinics to avoid unnecessary infection risks in diabetes clinics, but also considering need for referral of patients to specialised diabetes care and ways to continue chronic diabetes care after completion of TB treatment. The first international guidebook on combined TB and diabetes provides guidance on many of these issues. With regard to control of diabetes-associated TB at a population level, besides diabetes prevention (that will have huge impact beyond TB), TB preventive therapy among people with diabetes and LTBI is now being explored. To address some of the outstanding epidemiological and clinical questions on TB and diabetes (Table 4) we clearly require more study, including randomised clinical trials.

## Figures and Tables

**Table 1:** Key Issues Related to Screening TB Patients for Diabetes and Diabetes Patients for TB.

Screening TB patients for DM	Screening can identify many people with undiagnosed or diagnosed but poorly managed DM in many LMIC Blood glucose or HbA1c screening are not widely available in TB clinics in many LMIC; other screening tests are less accurate Transient (inflammation-related) hyperglycaemia which may fall with TB treatment; patients with newly identified hyperglycaemia should be tested again later in treatment Pathways for continued DM care at the end of TB treatment may be patchy and not always available, affordable, or high quality
Screening DM patients for TB	Practical difficulties with testing in DM clinics (e.g., availability of sputum smear and culture, CXR) Low yield, probably not cost-effective except if TB incidence very high
Screening DM patients for LTBI	No direct evidence assessing risks, benefits or cost-effectiveness of screening and treating LTBI in DM patients at present Expected difficulties with scale-up and quality control including exclusion of active TB disease

DM, diabetes; CXR, chest X-ray; LMIC, low- and middle-income countries.

**Table 2:** Key Issues Related to Treatment of Combined Diabetes and Tuberculosis.

TB treatment	As DM is associated with worse TB treatment outcomes, TB treatment may have to be prolonged or intensified and TDM should be considered DM is associated with TB drug resistance, suggesting that DST should be done for all TB-DM patients if this is no universal practice Drug interactions between rifampicin and drugs commonly used by people with DM TB-DM patients may experience more side-effects and drug-toxicity during TB treatment Polypharmacy may interfere with adherence and correct drug intake Many of these issues are especially relevant for HIV-infected patients
Glycaemic control	Glycaemic control is often difficult to achieve in combined TB-DM Metformin preferred oral drug; may even improve TB outcomes Insulin is indicated for severe hyperglycaemia (HbA1c > 10%) Patient and health systems factors guide choice and dosing of glucose-lowering drugs
Cardiovascular disease (CVD)	DM strongly associated with CVD; TB may further increase CVD risk There are huge gaps in CVD risk assessment and management among people with DM, and likely also among people with combined TB-DM Patients with combined TB-DM who have established CVD should receive antiplatelet, anti-hypertensives and lipid-lowering drugs as indicated Evidence is scare and more data are needed, also regarding lifestyle interventions and best care after completion of TB treatment

DM, diabetes; TDM, therapeutic drug monitoring; DST, drug susceptibility testing.

**Table 3:** Potential Strategies for Control of Diabetes-Associated Tuberculosis.

Strategy	Pro	Con
DM screening among TB patients	Possible improvement of individual patient outcome	No expected population impact
Better management of combined TB and DM	Possible improvement of individual patient outcome	No expected population impact
A more effective TB vaccine	Massive impact, also beyond DM-associated TB	Not available yet
Better DM management	Massive impact on all DM complications and mortality, especially related to cardiovascular disease	Challenging, especially in low-resource settings
TB preventive therapy for people with DM and LTBI	Projected reduction of individual TB risk, with significant population impact in terms of TB control	No evidence from phase 3 trials yet
Prevent diabetes incidence	Potentially massive impact on population health, CVD risk and saving substantial health care resources	Requires substantial shift in population diet and reductions in obesity. Limited evidence how to scale up to whole populations in LMIC

DM, diabetes; LTBI, latent tuberculosis infection; CVD, cardiovascular disease.

**Table 4:** Some Outstanding Clinical and Population Health Questions Related to Diabetes-Associated TB.

Are there any differences in risk of TB disease or treatment outcomes between people with type 1 and type 2 diabetes?
Is intermediate hyperglycaemia (“pre-diabetes”) a risk for TB disease and death?
Does diabetes (type 1, type 2) or intermediate hyperglycaemia affect the risk of LTBI?
What are the costs, risks/benefits of preventive therapy for LTBI, for individuals and populations?
How can health systems be organised to strengthen multi-morbidity care and prevention, e.g., through strengthening of primary care?
How should routine medications to reduce CVD risk be used in patients with TB and DM, and when should they be introduced?
Which anti-DM medications should be used in TB patients? How and when should they be introduced and adjusted for dose?
Should TB treatment be intensified or prolonged in people with diabetes?
How can smoking cessation/relapse prevention interventions be incorporated into TB-DM management?
Do TB survivors with diabetes face long term sequelae and higher risks, even after successful treatment completion (e.g., TB recurrence, cardiovascular or pulmonary disease or poor quality of life/disability)? How should they be followed up after completion of treatment?
How can the TB community advocate for investment in diabetes prevention and management?

## Data Availability

Not applicable.
